# Cognitive Demands and Memory-Related Outcomes of Emotion Regulation Tactics Among Younger and Older Adults

**DOI:** 10.1093/geroni/igaf122.048

**Published:** 2025-12-31

**Authors:** Hannah Wolfe, Derek Isaacowitz

**Affiliations:** Stockton University, Galloway, New Jersey, United States; Washington University in St. Louis, St. Louis, Missouri, United States

## Abstract

Older adults “paradoxically” report high emotional wellbeing despite declines in some cognitive abilities. The selection, optimization, and compensation with emotion regulation model (SOC-ER) suggests that older adults compensate for losses in cognitive resources by instead selecting and optimizing less cognitively-demanding (but effective) emotion regulation strategies. However, empirical evidence regarding which regulation behaviors are more cognitively demanding across the lifespan is limited. This study aimed to test the SOC-ER model by directly measuring the cognitive demand and outcomes of emotion regulation tactics. Younger (ages 18-25, N = 77) and older adults (ages 65+, N = 62) viewed blocks of negative images while instructed to use one regulation tactic per block: acceptance, positive-approaching, negative-receding, and negative-approaching. Cognitive demand was assessed for each tactic via self reports and pupillometry (a physiological measure of real time cognitive effort). Incidental memory for the stimuli was also assessed; participants completed a recognition memory test for all images presented during the regulation blocks. Results showed that while acceptance was self-reported as the least mentally demanding tactic and positive-approaching as the most, F(3,381)=3.655, p<.05, there was no significant difference in pupil dilation across tactics, F(3,351)=0.476, p=.699, ηp2=.004. Furthermore, positive-approaching was associated with the highest memory accuracy and negative-receding with the lowest, F(3,408)=13.90, p<.001, ηp2=.093. All interactions with age group were nonsignificant. Overall, the SOC-ER model was minimally supported, suggesting that older adults’ emotion regulation behaviors are not primarily driven by cognitive demand concerns.

